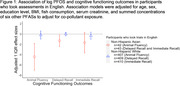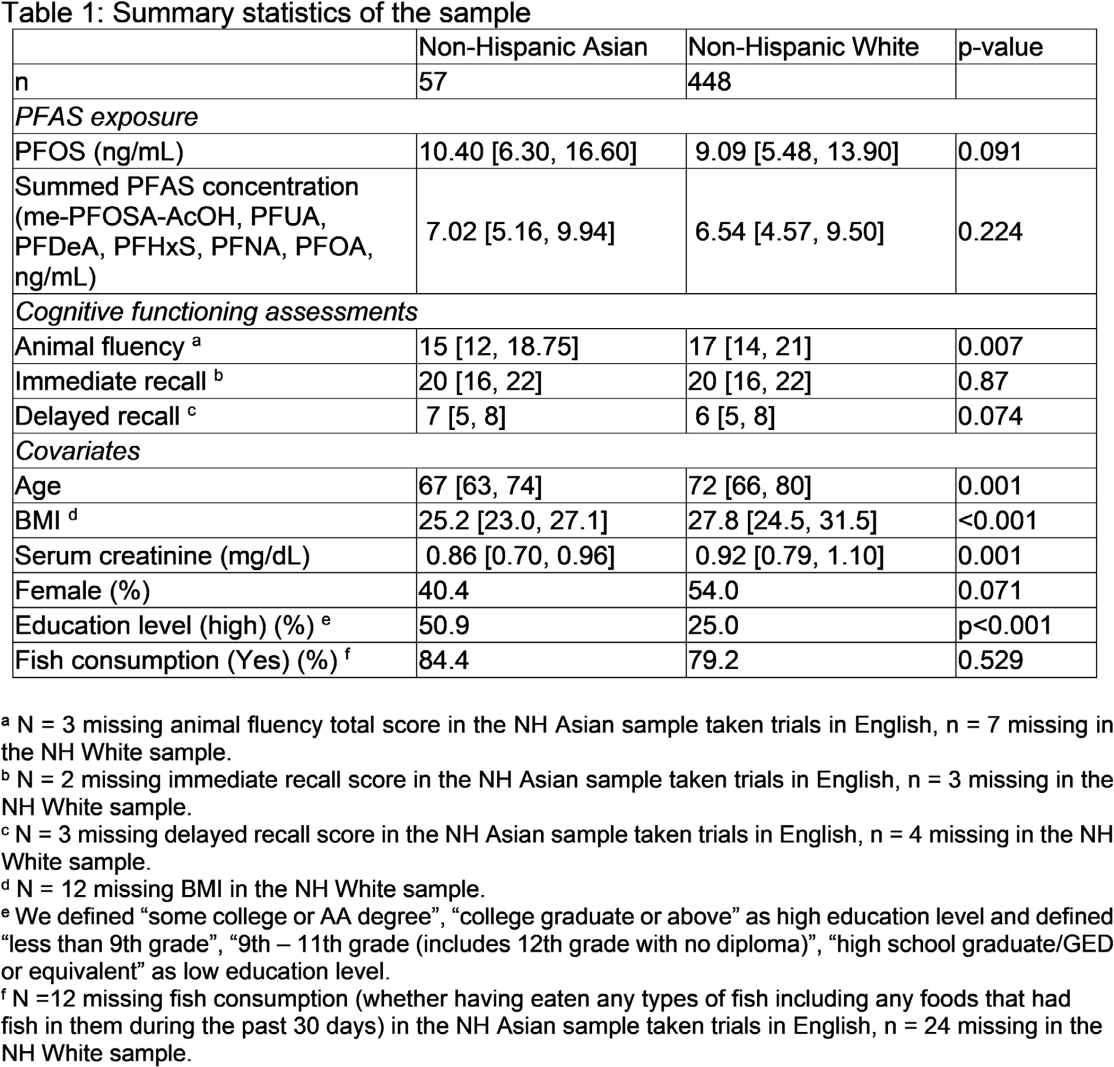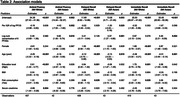# Exposure to perfluoroalkyl substances as a novel risk factor for cognitive impacts in older Asian Americans

**DOI:** 10.1002/alz.088182

**Published:** 2025-01-09

**Authors:** Shelley Liu, Yitong Chen, Katherine Manz, Jessie Buckley, Ankit Parekh, Carolyn W. Zhu, Jessica Spat‐Lemus, Walter A. Kukull, Mary Sano, Clara Li

**Affiliations:** ^1^ Icahn School of Medicine at Mount Sinai, New York, NY USA; ^2^ University of Michigan, Ann Arbor, MI USA; ^3^ University of North Carolina ‐ Chapel Hill, Chapel Hill, NC USA; ^4^ Icahn School of Medicine, Mount Sinai Hospital, New York, NY USA; ^5^ Department of Psychology, Montclair State University, Montclair, NJ USA; ^6^ National Alzheimer’s Coordinating Center, University of Washington, Seattle, WA USA; ^7^ Department of Psychiatry, Icahn School of Medicine at Mount Sinai, New York, NY USA

## Abstract

**Background:**

Environmental pollutants, called perfluoroalkyl substances (PFAS) have been linked to adverse cardiometabolic outcomes, immune dysfunction and cancer risk, but their associations with adult cognition are unknown. Nearly everyone in the United States has detectable levels of PFAS in their blood, but our prior work found that Asian Americans have the highest exposure burden. As Asian Americans fastest growing segment of older adults, examination of relationships between serum PFAS concentrations and cognition in Asian Americans urgently needed. Here, our study examined those associations in older Asian Americans using the National Health and Nutrition Examination Survey (NHANES), and compared findings to a non‐Hispanic White sample.

**Method:**

Our cross‐sectional study used 2011‐2014 (NHANES) data and included 57 non‐Hispanic Asian and 448 non‐Hispanic White participants who took the cognitive assessments in English, to minimize impact of assessment language on scores. Measures included the Animal Fluency test total score and word learning and recall modules from the Consortium to Establish a Registry for Alzheimer’s disease (CERAD) (immediate recall and delayed recall scores).

We used linear regression to estimate impact on cognitive functioning measures per interquartile (IQR) increase in log PFOS concentrations adjusting for age, sex, education, BMI, fish consumption, and serum creatinine. To adjust for co‐pollutant confounding, we also adjusted for summed concentration of other six other PFASs. We stratified all analyses by race/ethnicity group.

**Result:**

Our sample of Asian Americans had median age of 67 [IQR: 63, 74], and 41% were female. The non‐Hispanic White sample had median age of 72 [66, 80] and 54% were female. In Asian Americans, an IQR increase in log PFOS was associated with ‐4.42 (95% CI: ‐7.02, ‐1,82, p = 0.002) fewer words listed in the animal fluency test, ‐1.30 (95% CI: ‐2.43, ‐0.18, p = 0.025) words listed in the delayed recall, and ‐1.67 (95% CI: ‐3.68, 0.33, p = 0.098) fewer words listed in the immediate recall test (**Table 2** and **Figure 1**). All associations were null for non‐Hispanic Whites.

**Conclusion:**

Higher blood levels of perfluoroalkyl substances were associated with lower animal fluency scores and delayed recall scores in Asian Americans, but these associations were null for non‐Hispanic Whites.